# Relationship between Macrophage and Radiosensitivity in Human Primary and Recurrent Glioblastoma: In Silico Analysis with Publicly Available Datasets

**DOI:** 10.3390/biomedicines10020292

**Published:** 2022-01-27

**Authors:** Bum-Sup Jang, In Ah Kim

**Affiliations:** 1 Department of Radiation Oncology, Seoul National University Bundang Hospital, Seongnam 13620, Korea; bigwiz83@snu.ac.kr; 2 Department of Radiation Oncology, College of Medicine, Seoul National University, Seoul 03080, Korea

**Keywords:** glioblastoma, radiation, radiosensitivity index, macrophage, tumor microenvironment

## Abstract

The glioblastoma microenvironment predominantly contains tumor-associated macrophages that support tumor growth and invasion. We investigated the relationship between tumor radiosensitivity and infiltrating M1/M2 macrophage profiles in public datasets of primary and recurrent glioblastoma. We estimated the radiosensitivity index (RSI) score based on gene expression rankings. Macrophages were profiled using the deconvolution algorithm CIBERSORTx. Samples from The Cancer Genome Atlas (TCGA), Chinese Glioma Genome Atlas (CGGA), the Ivy Glioblastoma Atlas Project dataset, a single-cell RNA sequencing dataset (GSE84465), Glioma Longitudinal Analysis Consortium (GLASS), and an immunotherapy trial dataset (GSE121810) were included. RSI-high radioresistant tumors were associated with worse overall survival in TCGA and CGGA than RSI-low tumors. M1/M2 macrophage ratios and RSI scores were inversely associated, indicating that radioresistant glioblastoma tumor microenvironments contain more M2 than M1 macrophages. In the single-cell RNA sequencing dataset, the mean RSI of neoplastic cells was positively correlated with high M2 macrophages proportions. A favorable response to programmed cell death protein 1 (PD-1) therapy was observed in recurrent glioblastomas with high M1/M2 macrophage ratios and low RSI scores. In patients with recurrent glioblastoma, fewer M2 macrophages and low RSI scores were associated with improved overall survival. High M2 macrophage proportions may be involved in radioresistant glioblastoma.

## 1. Introduction

Checkpoint blockade immunotherapy has demonstrated remarkable success in several types of solid tumors. However, a recent trial of immune checkpoint blockades for recurrent glioblastoma, CheckMate 143 [1], showed limited efficacy. Compared with bevacizumab alone, only 8% of study patients showed an objective response in the CheckMate 143 trial, which may be explained by T-cell exhaustion in the tumor microenvironment [2]. The majority of immune cells in glioblastoma tumors consist of microglia and macrophages rather than T cells [3,4]. Thus, more research [5,6,7,8,9,10,11] has focused on targeting macrophages for the suppression of glioblastoma growth and relapse. In GBM, monocytic myeloid-derived suppressor cells have immune-suppressive functions including induction of T-cell apoptosis or expansion of regulatory T cells. Macrophage polarized into having this phenotype is defined as M2 macrophage [12]. M2 macrophages are known to accumulate in higher-grade gliomas [13,14]. Macrophage can also be polarized into the opposite phenotype to M2, which refers to M1 macrophage. M1 macrophages are associated with the release of pro-inflammatory cytokines and the promotion of antitumor immune responses [3]. Favorable or unfavorable immune microenvironment in glioblastoma may depend on polarized status in macrophage phenotype between M1 and M2. Here, we investigated the proportions of M1 and M2 macrophages within the tumor microenvironment based on publicly available glioblastoma transcriptome datasets using in silico methods.

Radiation therapy is effective for glioblastoma, and most patients receive radiotherapy during their treatment course. As the standard treatment, the concurrent administration of temozolomide is expected to provide a radiosensitizing effect that enhances the ability of radiation to kill tumor cells. However, glioblastomas are radioresistant tumors, and disease recurrence is almost inevitable. To quantify radiosensitivity, a radiosensitivity index (RSI) was developed based on the survival of NCI-60 human tumor cell lines after treatment with 2 Gy [15]. The RSI has been investigated and validated in several solid tumors, including breast cancer [16,17], pancreatic cancer [18], and glioblastoma [19]. The RSI was also integrated into a genomics-adjusted radiation dose model [20] to predict the time to first recurrence and overall survival in cancer patients who receive radiation therapy. With advances in immunotherapy, RSI has emerged as a potential biomarker for both radiation therapy and immunotherapy [21]. We hypothesized that the macrophage profile and RSI score of the immune microenvironment might determine treatment response and prognosis in glioblastoma.

Here, we collected datasets from publicly available glioblastoma transcriptome repositories, which were generated by various published studies. These included bulk tumor RNA sequencing datasets from primary and recurrent glioblastoma, as well as single-cell RNA sequencing datasets from primary glioblastoma and immune cells isolated from primary and recurrent human glioblastoma microenvironments. We then investigated the relationship between M1 and M2 macrophage profiles and radiosensitivity, represented as RSI scores, and their impacts on survival in patients with glioblastoma.

## 2. Materials and Methods

### 2.1. Data Sources

In this study, we collected and analyzed publicly available bulk and single-cell RNA sequencing datasets. To verify the predictive value of RSI, we acquired gene expression matrix and clinical datasheets from The Cancer Genome Atlas (TCGA, https://portal.gdc.cancer.gov (accessed on 6 January 2021)) and the Chinese Glioma Genome Atlas (CGGA, http://cgga.org.cn (accessed on 6 May 2020)). The Ivy Glioblastoma Atlas Project (https://glioblastoma.alleninstitute.org (accessed on 1 April 2021)) includes RNA sequencing data from seven anatomic microenvironment structures (MES) in the tumor microenvironment: cellular, hyperplastic, infiltrating, leading-edge, microvascular, perinecrotic, and pseudopalisading, which were identified by hematoxylin and eosin staining and dissected by laser. Detail definition of each microenvironment structure is described in the white paper in the Ivy Glioblastoma Atlas Project (https://help.brain-map.org/display/glioblastoma/Documentation (accessed on 1 April 2021)). For analysis, we categorized these MES into four MES: cellular MES were defined as within-tumor sites; hyperplastic and microvascular MES were defined as vascular sites; infiltrating and leading-edge were defined as peri-tumoral sites; and perinecrotic and pseudopalisading MES were defined as necrotic sites. Both a gene expression omnibus (GEO) study (identifier: GSE84465) and the brain immune atlas dataset (https://www.brainimmuneatlas.org (accessed on 8 May 2021)) were employed in the single-cell RNA sequencing analysis. The data included in GSE84465 were obtained from a study that performed single-cell RNA-seq in infiltrating neoplastic human glioblastoma cells [22]. The brain immune atlas includes the single-cell RNA sequencing datasets generated by the Movahedi lab across three publications [23,24,25]. Based on this dataset, Pombo et al. [24] profiled myeloid cells in glioblastoma across species and disease stages, and we adopted and analyzed the profile generated by Pombo et al. Next, the Glioma Longitudinal AnalySiS (GLASS) Consortium generated whole-genome, whole-exome, and RNA sequencing data in a time-series manner [26] (https://github.com/fpbarthel/GLASS (accessed on 15 May 2021)). From the GLASS dataset, we obtained publicly available paired RNA sequencing datasets: primary glioblastoma and the first recurrent episode. Clinical data were archived and evaluated considering RSI and M2 macrophage status. High RSI group was defined by upper third of RSI, and high- vs. low-M2 group was defined by its median relative abundance. To assess the outcomes of immunotherapy, we used another GEO study (identifier: GSE121810), which tested the efficacy of anti-programmed death-ligand 1 (PD-L1) blockade in recurrent glioblastoma.

### 2.2. RSI Calculation RSI and Macrophage Profiling

The RSI comprises 10 genes: *AR*, *JUN*, *STAT1*, *PRKCB*, *RELA*, *ABL1*, *SUMO1*, *CDK1*, *HDAC1*, and *IRF1*. We calculated the RSI score using a linear equation described in a previous study [21]. Using a rank-based linear regression model, we ranked these 10 genes according to their expression levels, with 10 being the highest and 1 being the lowest expression levels. RSI is a continuous value, and tumors presenting higher RSI scores are more radioresistant.

Immune cells were estimated using in silico methods based on the CIBERSORTx algorithm developed by Newman et al. [27]. CIBERSORTx can profile cell types of interest, based on gene expression, providing relative cell proportions from mixed cell populations or bulk tumor analyses. Using the web-based interactive user interface (https://cibersortx.stanford.edu (accessed on 1 June 2021)), we profiled immune cells based on the leukocyte gene signature matrix (LM22). M1 and M2 macrophage were determined by imputation of LM22 gene signature expression profiles by using the CIBERSORTx platform. The CIBERSORTx was applied to single-cell RNA sequencing datasets, treating every single cell as one bulk sample. The single cell was classified according to the immune cell type showing the highest fraction. CIBERSORTx also offers a gene expression module by the group level, which enables the imputation of a transcriptome profile for each cell type. After gene expression was imputed for each cell, we identified differentially expressed genes between groups and performed gene set enrichment analysis using the TCGAbiolinks [28] R package. The M1/M2 macrophage ratio was calculated by dividing M1 macrophage fractions by M2 macrophage fractions.

### 2.3. Statistical Analysis

Available survival data were analyzed with the Cox proportional hazards model. A Kaplan–Meier curve was plotted, and the log-rank test was performed. A simple linear regression model was adopted to reveal the correlation between RSI scores and the M1/M2 macrophage ratio. For paired samples, we performed the Wilcoxon signed-rank test using PRISM software version 9 (GraphPad Software, Inc, San Diego, CA, USA). Two-sided *p*-values were estimated. Heatmaps were plotted using the ‘ComplexHeatmap’ [29] R package. Bar graphs and pie charts were plotted using PRISM software. Sankey plots and scatter plots were generated by Microsoft Power BI Desktop software.

### 2.4. Ethics Statement

All the information of patients was obtained from publicly available datasets. All the patients and treatments were complied with the principles laid down in the Declaration of Helsinki in 1964 and its later amendments or comparable ethical standards.

## 3. Results

### 3.1. Radiosensitivity Index Was Predictive for Overall Survival Response to Radiation Therapy

**Figure 1:**
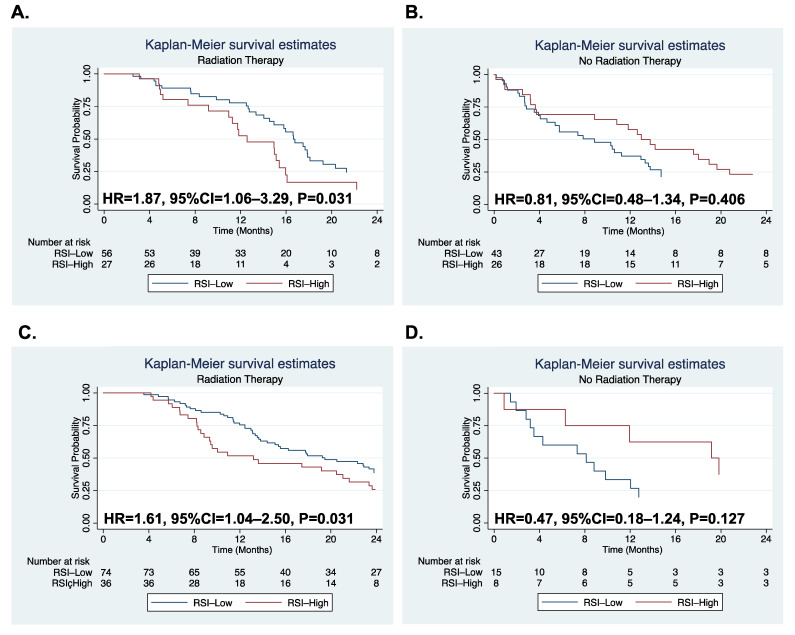
In TCGA glioblastoma population, Kaplan–Meier curves are compared between RSI-high and RSI-low groups for patients who received radiation therapy (**A**) and did not receive it (**B**). In CCGA glioblastoma population, Kaplan–Meier curves are compared between RSI-high and RSI-low groups for patients who received radiation therapy (**C**) and did not receive it (**D**). *p*-value was estimated by Cox proportional hazard model. Abbreviations: HR, hazard ratio; CI, confidence interval; RSI, radiosensitive index; TCGA, The Cancer Genome Atlas, CGGA, The Chinese Glioma Genome Atlas.

We collected publicly available glioblastoma data from two large databases: TCGA (N = 152) and CGGA (N = 133). First, we populated the distribution of RSI scores in each dataset and found a bimodal distribution and a unimodal distribution in the TCGA and CGGA datasets, respectively (Supplementary Figure S1). The cutoff values for RSI were determined to be 0.88 for TCGA and 0.47 for CGGA. Of glioblastoma, these cutoff values grouped irradiated patients into RSI-high (33%) and RSI-low (67%) groups in both the TCGA and CGGA datasets. In the TCGA dataset, RSI-high was associated with significantly reduced survival compared with RSI-low among patients who received radiation therapy (Figure 1A, hazard ratio (HR) = 1.87, 95% confidence interval (CI) = 1.06–3.29, *p* = 0.031). However, no significant difference in survival was observed between RSI-high and RSI-low groups among patients who did not receive radiation therapy (Figure 1B, HR = 0.81, 95% CI = 0.48–1.34, *p* = 0.406). In the CGGA dataset, similar to the TCGA, the RSI-high group demonstrated worse survival than the RSI-low group among patients receiving radiation therapy (Figure 1C, HR = 1.61, 95% CI = 1.04–2.50, *p* = 0.031). However, no significant survival difference was observed between the RSI-high and RSI-low groups among patients who did not receive radiation therapy (Figure 1D, HR = 0.47, 95% CI = 0.18–1.24, *p* = 0.127). These results indicate that RSI is predictive for overall survival among patients who are treated with radiation therapy.

### 3.2. High RSI Was Associated with a Low M1/M2 Macrophage Ratio

**Figure 2:**
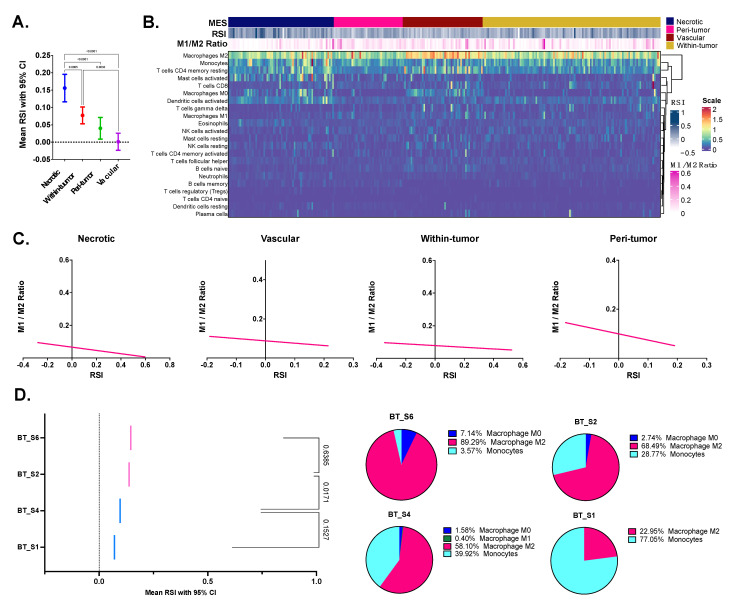
(**A**) In the Ivy Glioblastoma Atlas Project dataset, RSI was compared between anatomic MES in the tumor microenvironment. *p*-values, estimated by one-way analysis of variance (ANOVA), are shown in the graph. (**B**) Heatmap representing immune cells deconvoluted by the CIBERSORTx algorithm and RSI according to anatomic MES. (**C**) Scatter plots showing RSI and M1/M2 macrophage ratio according to anatomic MES. Fitted line (sold) and its 95% CI error (dotted) is shown in each plot. (**D**) In a single-cell RNA sequencing dataset (GSE84465), mean RSI with 95% CI are estimated from collection of single cells in each study patient. *p*-value was estimated by ANOVA. In the right panel, pie charts are demonstrated with respect to proportion of macrophages. Abbreviations: CI, confidence interval; M1, M1 macrophage; M2, M2 macrophage; RSI, radiosensitive index; MES, microenvironment structures.

The Ivy Glioblastoma Atlas Project is a publicly available repository created to explore the anatomic and genetic bases of glioblastoma at the cellular and molecular levels. We calculated mean RSI scores according to microenvironment structure and found that the mean RSI score for the necrotic microenvironment structure was significantly higher than those for other MES (Figure 2A). High mean RSI was also observed for the within-tumor microenvironment structure. The lowest mean RSI score was identified for the vascular microenvironment structure. These results demonstrated that radioresistant cells were located in the necrotic and within-tumor MES, whereas the vascular microenvironment structure had a radiosensitive environment. To investigate the immune microenvironment according to the microenvironment structure, we profiled immune cells using the CIBERSORTx algorithm. As shown in the heatmap in Figure 2B, we identified a pattern of high RSI scores in the necrotic and within-tumor MES. In addition, differential patterns of low M1/M2 macrophage ratios were also found at each microenvironment structure. Next, we examined the relationships between the M1/M2 ratio and the RSI score for each microenvironment structure (Figure 2C). Negative correlations were found at all MES, as assessed by the linear regression slope: −0.10 for the necrotic microenvironment structure, −0.25 for the peri-tumoral microenvironment structure, −0.12 for the vascular microenvironment structure, and −0.05 for the within-tumor microenvironment structure. A significant negative relationship was only found in the peri-tumoral microenvironment structure (*p* = 0.017). In the single-cell RNA sequencing dataset (GSE844655), four patient samples were investigated by calculating their mean RSI scores and profiling immune cells using CIBERSORT (Figure 2D). The mean RSI scores were distributed as follows, from highest to lowest: BT_S6, BT _S2, BT_S4, and BT_S1 from radioresistant to radiosensitive. This same distribution trend was observed for the proportions of M2 macrophages; 89.29% for BT_S6, 68.49% for BT_S2, 58.10% for BT_S4, and 22.95% for BT_S1 (Figure 2D). Therefore, more M2 macrophages in certain tumor areas were associated with a more radioresistant tumor.

### 3.3. RSI and Macrophage Profile between Primary and Recurrent Glioblastoma

For bulk tumor samples, we used the GLASS Consortium dataset, consisting of glioma tumor specimens sequenced in a time-series manner. Among the multiple GLASS datasets, we selected matched bulk RNA sequenced samples from primary and recurrent glioblastoma occurrences (N = 32 pairs). As shown in Supplementary Figure S2, The RSI scores and M1/M2 macrophage ratios did not differ between paired samples. However, we observed lower median M1/M2 macrophage ratios in recurrent glioblastoma compared with primary glioblastoma (median difference = −0.000537, Wilcoxon signed-rank test, *p* = 0.7190).

**Figure 3:**
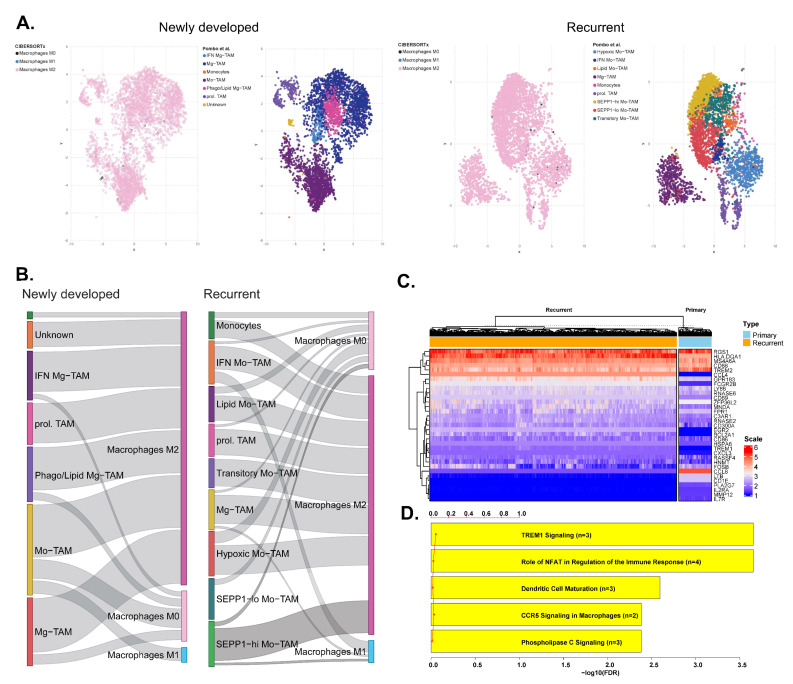
From the brain immune atlas dataset, immune cells classified by CIBERSORTx and those reported by Pombo et al. [24] are compared for newly developed and recurrent glioblastoma in scatter plots (**A**) and a Sankey diagrams (**B**). (**C**) Heatmap showing macrophage gene expression patterns between primary and recurrent glioblastoma from the brain immune atlas dataset. (**D**) Gene set enrichment analysis for differential genes of macrophage between primary and recurrent glioblastoma. Abbreviations: TAM, tumor-associated macrophage; FDR, false discovery rate.

For single-cell-level analyses, we collected single-cell RNA sequencing data from the brain immune atlas. In immune cells isolated from human glioblastoma, we adopted the classification data reported by Pombo et al. [24]. We then re-classified those immune cells into 22 human hematopoietic cell phenotypes using the CIBERSORTx algorithm. Finally, we compared both classification for macrophages in both primary and recurrent glioblastoma cases. Strikingly, as shown in Figure 3A, we found that nearly all macrophages classified by Pombo et al. were classified as M2 macrophages in both primary and recurrent glioblastoma cases. For M1 macrophages, we plotted a Sankey diagram between classifications (Figure 3B). In primary glioblastoma cases, M1 macrophages were classified by CIBERSORT as monocyte-derived tumor-associated macrophages (Mo-TAMs), based on the expression of genes such as *TGFB1*, *CLEC12A*, and *FXYD5* [24]. In recurrent glioblastoma cases, the majority of M1 macrophages were classified as interferon (IFN) Mo-TAMs, which are Mo-TAMs with IFN-induced signatures. A small portion of M1 macrophages were identified as microglial tumor-associated macrophages (Mg-TAMs), which express microglial signature genes. A minority of M1 macrophages were classified as SEPP-hi-Mo-TAMs, which are characterized by the low expression of microglial genes and the high expression of *SLC40A1*, *FOLR2*, *MRC1*, and *RNASE1*, which are genes associated with anti-inflammatory activation [24]. Using the CIBERSORTx group analysis method, we sought to impute cell type-specific gene expression from single-cell RNA sequencing data. Imputed gene expression was then compared between primary and recurrent glioblastoma cases. Several macrophage-related genes, including *TREM1*, *TREM2*, *CCL4*, *CCL8*, or *CXCL3*, showed differential patterns between the two groups (Figure 3C). After differential genes were identified, gene set enrichment analysis demonstrated that the triggering receptor expressed on myeloid cells 1 (TREM1) signaling pathway was significantly different between macrophages associated with primary and recurrent glioblastoma (Figure 3D, false-discovery rate (FDR) < 0.001). In addition, other pathways relevant to macrophage signaling, such as nuclear factor of activated T cells (NFAT) activity (FDR < 0.001), C-C chemokine receptor type 5 (CCR5) signaling (FDR = 0.004), and phospholipase C signaling (FDR = 0.004), were also found to be different between the two groups. These results showed differential macrophage activities between primary and recurrent glioblastoma, although most macrophages presented as M2 macrophages.

### 3.4. RSI and Macrophage Profile Were Associated with Treatment Response in Recurrent Glioblastoma

**Figure 4:**
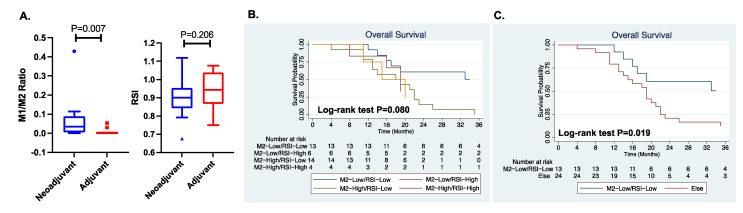
(**A**) From an immunotherapy trial dataset (GSE121810), the ratio of M1/M2 macrophage and the mean value of RSI in neoadjuvant and adjuvant arm are compared. *p*-value was estimated with analysis of variance. (**B**) From the Glioma Longitudinal Analysis Consortium (GLASS), overall survival for recurrent glioblastoma population is compared, stratified by M2 macrophage and RSI status. *p*-value was estimated by log-rank test. (**C**) Comparison of overall survival between M2 macrophage-low and RSI-low group versus other population in recurrent glioblastoma. Abbreviations: M1, M1 macrophage; M2, M2 macrophage; RSI, radiosensitive index.

To investigate how treatment response relates to RSI scores and macrophage profiles, we used a publicly available dataset (GSE121810), which was generated from a neoadjuvant PD-1 blockade trial for recurrent glioblastoma. Cloughesy et al. [30], who provided the data, revealed that the neoadjuvant administration of PD-1 blockades enhances local/systemic antitumor immune responses and patient survival. From the available dataset, we analyzed 14 patients who received PD-1 blockade therapy in the neoadjuvant setting and 15 patients who received PD-1 blockade therapy in the adjuvant setting. As shown in Figure 4A, a lower M1/M2 macrophage ratio was observed in the adjuvant setting than in the neoadjuvant settings (*p* = 0.007). However, the RSI scores were not significantly different between the two treatment settings. From the GLASS Consortium clinical dataset, we explored the prognostic impacts of RSI scores and macrophage profiles for overall survival in patients with recurrent glioblastoma. The cutoff for the M2 macrophage proportion was determined by the median value, and the cutoff for the RSI score was selected to represent the upper third of the population, similar to the cutoff value used for the TCGA and CGGA datasets. As shown in Figure 4B, patients with recurrent glioblastoma characterized by low M2 macrophage proportions and low RSI scores demonstrated improved survival. When we compared the M2-low/RSI-low group against the other groups, survival was significantly increased in the M2-low/RSI-low group (log-rank test *p* = 0.019).

## 4. Discussion

We demonstrated the prognostic impact of macrophage profiles and the predictive value of RSI scores in patients with glioblastoma. Single-cell and bulk tumor RNA sequencing datasets revealed the existence of a relationship between the proportions of M1 and M2 macrophages and RSI scores, as well as a positive correlation between radioresistant tumors and low M1/M2 macrophage ratios. Although RSI scores did not change between primary and recurrent tumors, differential macrophage gene expression was observed between these two tumor types. In recurrent glioblastoma, the M1/M2 macrophage ratio and the RSI score combined with the M2 macrophage proportion demonstrated the potential to serve as predictive markers for immunotherapy and salvage therapy, respectively.

Most glioblastoma patients receive radiation therapy during their disease course, and tumor radiosensitivity can determine the treatment response. Consistent with accumulating evidence supporting the validity of the RSI score [16,17,18,19], we used two large glioblastoma databases in the current study to verify the predictive value of the RSI score for the response to radiation therapy. RSI scores demonstrated prognostic power only among patients who received radiation therapy, which indicates its predictive value. Radiation therapy takes 1.5 months after 1–1.5 months from surgical resection. Radiation oncologists can evaluate radiation response early at 6 months after the end of radiotherapy. Additionally, six cycles of temozolomide were administered at 6 months after the end of radiotherapy. Altogether, roughly 10 months after surgical resection may be needed to evaluate radiation response. In the current study, 10-month overall survival in glioblastoma is 67.3% and 71.2% in TCGA and CGCA, respectively. Thus, we presumed that RSI upper one-third of patients in glioblastoma indicated dead patients with radioresistant glioblastoma. Some studies have suggested the use of a fixed RSI cutoff value. Strom et al. [31] collected gene expression data from 10,240 solid primary tumors and identified the local minimum in the bimodal RSI density plot. Radiosensitive RSI-low tumors were defined as having RSI values below 0.3745. By contrast, radioresistant RSI-high tumors had RSI values equal to or greater than 0.3745. However, this cutoff only classified 1.7% of brain glioma cases as RSI-low, whereas 98.3% were classified as RSI-high. Similarly, Dai et al. [21] used the cutoff RSI value of 0.46 for stratification into RSI-low and RSI-high groups. A total of 8386 samples from the Merged Microarray-Acquired dataset (MMD) and a total of 6116 primary tumor samples from TCGA were analyzed. However, their analyzed datasets did not include glioma samples. In this study, we focused on glioblastoma as the study population and revealed that roughly one-third of patients had radiosensitive tumors.

We hypothesized that RSI scores in glioblastoma might be related to the tumor microenvironment with respect to macrophage proportions. Generally, glioblastomas are considered immunologically cold tumors deficient in T-cell infiltration [32]. In several immunotherapy trials [1,33,34], checkpoint inhibitors, such as nivolumab or pembrolizumab, showed only modest effects in recurrent glioma. Immunotherapy agents target T-cell priming or activation, and the low infiltration of T cells is likely to contribute to the limited efficacy observed for these agents in glioblastoma. Myeloid cells are the most abundant cell type in the glioblastoma tumor microenvironment [35]. Tumor-associated macrophages, such as M2 macrophages, may contribute to immunosuppression and tumor progression [36]. Although the simple classification of macrophages into M1 and M2 types provides a conceptual framework for understanding the glioblastoma immune microenvironment, the differentiation of glioblastoma-associated macrophages represents a continuous process [37]. Thus, we used M1/M2 macrophage ratios as a surrogate marker for the immune status of the glioblastoma microenvironment. A low M1/M2 macrophage ratio represents an immune-suppressive tumor microenvironment. Across MES, RSI score and the ratio of M1/M2 macrophage demonstrated the trend of inverse correlation. Of note, a significant inverse relationship identified in the peri-tumoral microenvironment structure, where most resident microglia are located in glioblastoma [12]. At the single-cell level, patients featuring tumor cells with a higher mean RSI score also presented with an increased proportion of M2 macrophages. Consistent with these results, Strom et al. [31] identified that RSI scores and a 12-chemokine signature were related, and those with lower RSI scores showed higher immune activation. The 12-chemokine signature was developed as a surrogate marker of immune activation for intratumoral lymphoid cell aggregates; however, the study population characterized by Strom et al. did not include glioma patients. Dai et al. [21] revealed that lower RSI scores were associated with higher proportions of M1 macrophages in two large transcriptome databases. These findings verify the relationship between radiosensitivity and immune status, particularly with respect to the proportions of M1 and M2 macrophages in the glioblastoma microenvironment.

Using primary and recurrent glioblastoma samples from bulk and single-cell RNA sequencing datasets, the patterns of RSI scores and macrophage profiles were compared, and their impacts on overall survival were investigated. In bulk tumor samples from paired primary and recurrent glioblastoma cases, RSI scores did not change across time points. Compared with primary glioblastoma, the M1/M2 macrophage ratio decreased in recurrent tumors, but this change was not statistically significant. Using CIBERSORTx, we found that nearly all macrophages were characterized as the M2 type in both primary and recurrent samples. By contrast, Antunes et al. [24] revealed a dynamic heterogeneity among macrophages based on single-cell analysis. This difference in findings may be due to the design of CIBERSORTx for use on bulk transcriptomes. Nevertheless, we found that transcriptome status was altered in macrophages between primary and recurrent glioblastoma, including TREM1 signaling, NFAT activity, CCR5 signaling, and phospholipase C signaling. TREM1 signaling was differentially identified in radioresistant and high PD-L1-expressing glioblastoma tumors in the TCGA glioblastoma population [38]. Although TREM1 is considered to be a marker associated with M1 macrophages, high TREM1 expression is associated with poor survival among glioma patients [4]. Kong et al. [39] found that the expression of TREM1 had a positive relationship with M2 macrophages in the TCGA glioblastoma population. The in vitro upregulation of TREM1 in macrophages triggered the release of colony-stimulating factor-1 (CSF-1), triggering invasive activity and pathologic angiogenesis in glioblastoma cells. NFAT signaling is known to contribute to macrophage activation, and the pharmacological blockade of NFAT signaling reduces the incidence of glioblastoma recurrence in preclinical models [39]. A relationship between the CCR5 system and microglia polarization also appears to exist, as the pharmacological blockade of CCR5 prevented the polarization of M2 macrophages [39]. Together, these signaling pathways may determine macrophage phenotypes, leading to disease recurrence.

We also hypothesized that the M1 and M2 macrophage profile would be related to the treatment response, and tumor-associated macrophages [40,41] have been suggested as one potential mechanism for resistance to anti-angiogenic therapy in glioblastoma. We found that a favorable immune microenvironment induced by neoadjuvant PD-1 blockade was associated with a low M1/M2 macrophage ratio. The RSI score and macrophage profile impacted overall survival in patients who experienced a recurrence event during their disease course. Although RSI scores did not change between primary and recurrent glioblastoma events, the radiosensitivity and immune microenvironment of the initial tumor may be important. Wang et al. [13] observed no differences in the microenvironment between primary tumors with short- and long-term recurrence. However, they found that short-term relapse glioblastomas demonstrated a significantly higher fraction of M2 macrophages after radiation compared with long-term relapse tumors, suggesting that M2 macrophages may play a role in radioresistance. Several studies [7,8] suggested that immunotherapy targeting M2 macrophages may have a radiosensitizing effect. Thus, alleviating the M1/M2 ratio or converting M2 macrophages into M1 macrophages may represent a potential therapeutic approach for glioblastoma [42]. Several factors are involved in the attraction of TAMs to the tumor site, including CSF-1 receptor (CSF-1R) and granulocyte-macrophage colony-stimulating factor (GM-CSF) [3]. CSF-1R inhibitors are known to deplete or polarize TAMs, leading to the suppression of glioma progression [42]. Therefore, CSF-1R inhibitors are expected to modulate myeloid cells within the immune-suppressive glioma tumor microenvironment.

Given both the role of radiation therapy in glioblastoma and the relationship between radiosensitivity and the macrophage profile identified in this study, a combination of CSF-1R inhibitor and irradiation may represent an effective approach to glioblastoma therapy. The CSF-1R inhibitor BLZ-945 reduced M2 macrophages when radiation increased in an orthotopic immunocompetent glioblastoma model. Akkari et al. [10] found that CSF-1R inhibitor treatment combined with radiation therapy enhanced survival in preclinical models. In their experiment, CSF-1R inhibitor alone failed to suppress tumor growth, resulting in no overall survival advantage. However, combining radiation with a continuous CSF-1R inhibitor blocked the radiation-acquired phenotype of brain-resident microglia and monocyte-derived macrophages, which promoted a radiation response and led to the delay or prevention of recurrent disease. In a current clinical study, the combination of radiation and CSF-1R inhibitor is being tested in glioblastoma, and a Phase Ib/II study of combined PLX3397, radiation therapy, and temozolomide in patients with primary glioblastoma (NCT01790503) recently completed recruitment. The role of M1 and M2 macrophages with respect to radiosensitivity in glioblastoma should be studied experimentally. Since polarization of M1 and M2 macrophage was demonstrated in in vitro studies, their contribution to radiosensitivity can be revealed in an experiment setting. To reflect tumor/immune microenvironment, a glioblastoma organoid model may be needed.

## 5. Conclusions

RSI scores can predict the radiation response in terms of overall survival among patients with gli oblastoma. A high proportion of M2 macrophages may play an important role in the tumor microenvironment of radioresistant glioblastoma.

## Data Availability

All data analyzed during this study are available from the publicly available repository. Data from TCGA can be downloaded from the Genomic Data Commons Data Portal (https://portal.gdc.cancer.gov (accessed on 6 January 2021)) and CGCA from the website (http://cgga.org.cn (accessed on 6 May 2020)). Data for the Ivy Glioblastoma Atlas Project can be retrieved from the website (https://glioblastoma.alleninstitute.org (accessed on 1 April 2021)). Single-cell RNA sequencing data are available using identifier GSE84465 in the GEO database. The brain immune atlas is available from the website (https://www.brainimmuneatlas.org (accessed on 8 May 2021)). Paired bulk tumor RNA sequencing data were retrieved from the GLASS Consortium (https://github.com/fpbarthel/GLASS (accessed on 15 May 2021)).

## Supplementary Materials

The following supporting information can be downloaded at: https://www.mdpi.com/article/10.3390/biomedicines10020292/s1, Figure S1: Distribution of RSI scores in the TCGA and CGGA datasets; Figure S2: Comparison of M1/M2 macrophage ratios and RSI scores in paired primary and recurrent tumors.
