# Supplementary material for: Relationship between Macrophage and Radiosensitivity in Human Primary and Recurrent Glioblastoma: In Silico Analysis with Publicly Available Datasets
Source: Biomedicines. 2022 Jan 27;10(2):292. doi: 10.3390/biomedicines10020292 (PMC8869561; doi:10.3390/biomedicines10020292)
Supplement: Supplementary file 1 [file biomedicines-10-00292-s001.zip › biomedicines-1551970-Figure S2.pdf]

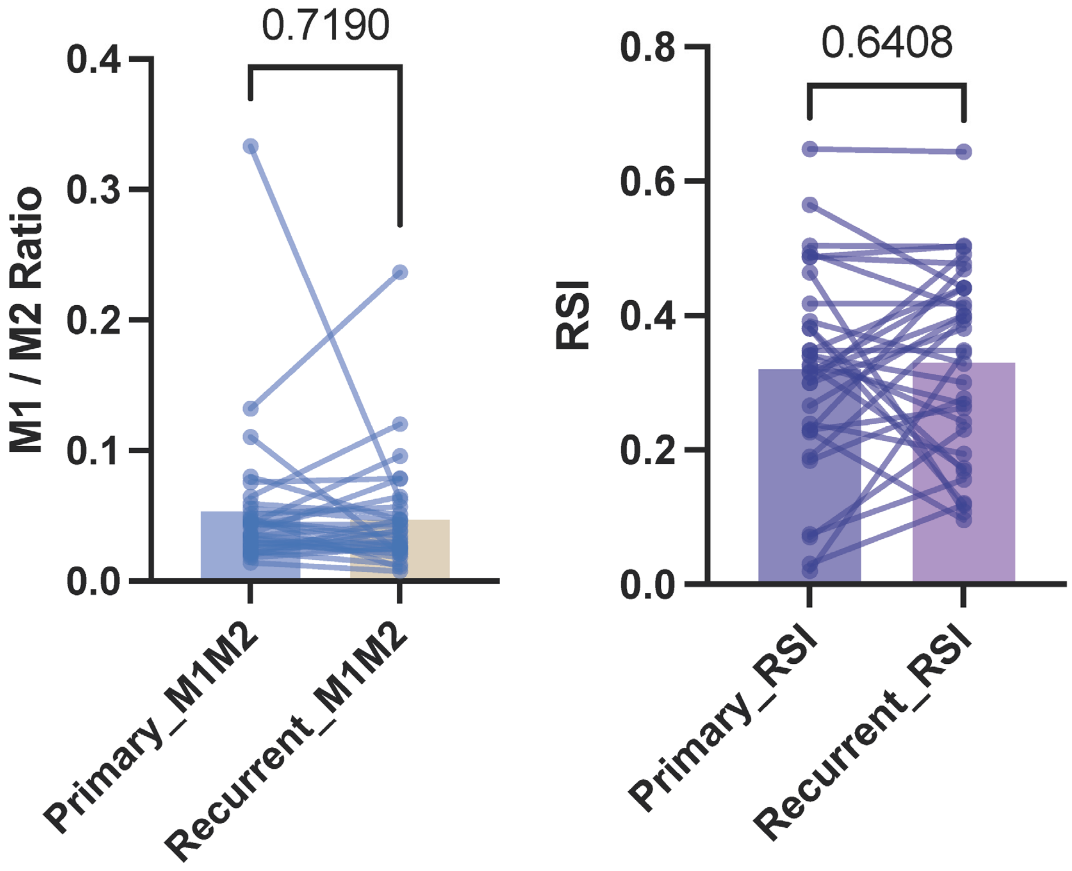

Figure S2. Comparison of M1/M2 macrophage ratios and RSI scores in paired primary and recurrent tumors.
