# Supplementary figures and images for: Relationship between Macrophage and Radiosensitivity in Human Primary and Recurrent Glioblastoma: In Silico Analysis with Publicly Available Datasets
Source: Biomedicines. 2022 Jan 27;10(2):292. doi: 10.3390/biomedicines10020292 (PMC8869561; doi:10.3390/biomedicines10020292)

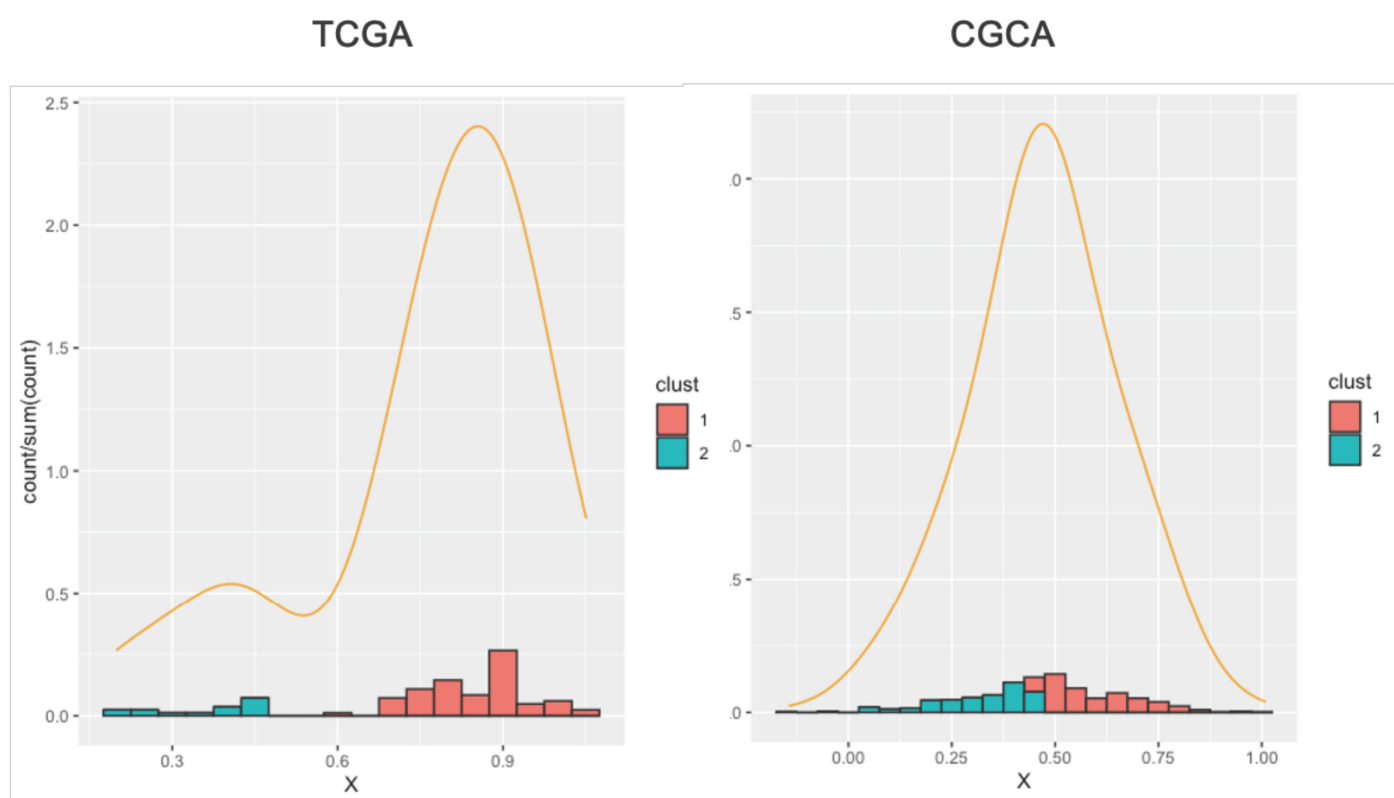

**Figure S1.** Distribution of RSI scores in the TCGA and CGGA datasets.

Supplement: Supplementary file 1 [file biomedicines-10-00292-s001.zip › biomedicines-1551970-Figure S1.pdf]
